# New VLBI2010 scheduling strategies and implications on the terrestrial reference frames

**DOI:** 10.1007/s00190-014-0697-9

**Published:** 2014-02-12

**Authors:** Jing Sun, Johannes Böhm, Tobias Nilsson, Hana Krásná, Sigrid Böhm, Harald Schuh

**Affiliations:** 1 Department of Geodesy and Geoinformation, Vienna University of Technology, Vienna, Austria; 2 National Key Laboratory of Science and Technology on Aerospace Flight Dynamics, Beijing Aerospace Control Center, Beijing, China; 3 GFZ German Research Centre for Geosciences, Telegrafenberg, 14473 Potsdam, Germany

**Keywords:** Very long baseline interferometry, Scheduling, TRF, CRF

## Abstract

In connection with the work for the next generation VLBI2010 Global Observing System (VGOS) of the International VLBI Service for Geodesy and Astrometry, a new scheduling package (Vie_Sched) has been developed at the Vienna University of Technology as a part of the Vienna VLBI Software. In addition to the classical station-based approach it is equipped with a new scheduling strategy based on the radio sources to be observed. We introduce different configurations of source-based scheduling options and investigate the implications on present and future VLBI2010 geodetic schedules. By comparison to existing VLBI schedules of the continuous campaign CONT11, we find that the source-based approach with two sources has a performance similar to the station-based approach in terms of number of observations, sky coverage, and geodetic parameters. For an artificial 16 station VLBI2010 network, the source-based approach with four sources provides an improved distribution of source observations on the celestial sphere. Monte Carlo simulations yield slightly better repeatabilities of station coordinates with the source-based approach with two sources or four sources than the classical strategy. The new VLBI scheduling software with its alternative scheduling strategy offers a promising option with respect to applications of the VGOS.

## Introduction

The very long baseline interferometry (VLBI) technique has now been employed in geodesy for over 40 years and plays an important role for the realization of global geodetic reference frames. In particular, it is a major contributor to the definition of a stable scale of the terrestrial reference frame (TRF) and has a unique capability to determine the celestial reference frame (CRF) and the orientation of the Earth in space, an essential element for all positioning and navigation applications. The current accuracy for VLBI station position determination from a 24-h observing session is on the level of 5 mm (Schuh and Behrend 2012). An important step towards that level of accuracy has been the development of scheduling algorithms that allow the successful separation of the various geodetic parameters in large multi-parameter adjustments (Steufmehl 1991, 1994).

Within the frame of International Association of Geodesy’s (IAG’s) key component Global Geodetic Observing System (GGOS), it has become clear that modern space geodetic techniques should provide station coordinates and/or baseline length time series with an accuracy better than 1 mm. Only then it will be possible to detect and study subtle effects like non-linear station motions related to geo-hazards such as earthquakes or long-term effects due to global changes such as sea level rise (Plag et al. 2009). All current VLBI systems and processes, from antennas to analyses, have been reviewed within the International VLBI Service for Geodesy and Astrometry (IVS). A path to the next-generation VLBI system [called VLBI2010, and its corresponding global network the VLBI2010 Global Observing System (VGOS)] with unprecedented new capabilities has been outlined (Niell et al. 2005). The three major goals are

- 1 mm station position and 0.1 mm/year station velocity accuracy on global scales;
- Continuous measurements for time series of station positions and Earth orientation parameters (EOP);
- Turnaround time to initial geodetic results of $<$24 h.

To fulfil the requirements of VLBI2010, various new facets have been investigated (Petrachenko et al. 2009a; Behrend et al. 2009; Petrachenko et al. 2009). A new VLBI system based on fast moving (12$^\circ$/s in azimuth and 6$^\circ$/s in elevation), small ($\approx$12 m diameter), mechanically stable antennas that can be replicated economically has been proposed. To shorten the on-source observing time, a four-band system is recommended that uses a broadband feed to span the entire frequency range from 2 to 14 GHz. For detecting an adequate number of high-quality radio sources, a total instantaneous data rate as high as 32 Gbps and a sustained data storage or transmission rate as high as 8 Gbps are necessary. It is expected that the important contribution that VLBI provides for the scale and orientation of global reference frames will be improved with a more uniform distribution of sites on the globe and with an increased number of sites in the southern hemisphere. The use of multiple antennas at a site is also proposed within VLBI2010. Up to summer 2013, a number of VLBI2010 projects are in progress: several antennas have been erected and construction of about ten more new antennas is at various stages of completion (Hase et al. 2012).

In the final report of IVS Working Group 3 “VLBI2010 (Niell et al. 2005), the importance of new observing strategies and scheduling algorithms was clearly recognized. Considering also the recommendations for future IVS products given in the final report of the former IVS Working Group 2 Product Specification and Observing Programs (Schuh et al. 2002), a new set of criteria to specify the next generation geodetic VLBI system was established within the IVS. At Goddard Space Flight Center (GSFC), Greenbelt, USA, the Sked software has been updated for VLBI2010 scheduling (Gipson 2012). Furthermore, a new scheduling package (Vie_Sched) has been developed at the Institute of Geodesy and Geophysics of the Vienna University of Technology. It is a part of the Vienna VLBI Software (VieVS) (Bohm et al. 2009), which is based on Matlab script files (www.mathworks.com). The new VLBI2010 scheduling package presented in this article includes new scheduling approaches, takes into considerations the present and the future VLBI2010 requirements, and implements the various specifications of the VLBI2010 antennas. The main goal of Vie_Sched is to exploit the advantages of the future VLBI2010 system and, in this context, to derive the highest accuracy for the geodetic parameters. Besides the conventional station-based scheduling strategy, a new source-based scheduling strategy for VLBI2010 was implemented in Vie_Sched.

This paper introduces in detail the new approach of source-based scheduling and the differences to the conventional station-based strategy. The different VLBI scheduling strategies and options are carefully compared and investigated and the implications on TRF are thoroughly evaluated based on Monte Carlo simulations.

## Conventional station-based scheduling strategy (abbreviated as SB)

The conventional station-based scheduling strategy is to achieve uniform sky coverage at each station, i.e. the sky coverage is optimized in short intervals taking into account the rapid atmospheric variability, partly at the expense of the total number of observations. In all cases of schedule optimization, the sky coverage is essential to de-correlate zenith wet delay (zwd), clock parameters, and station heights. However, it has never been clearly defined what a uniform sky coverage is. Some (weak) restrictions which guarantee a fairly uniform sky distribution are that nearby radio sources should not be observed within certain time intervals, and that the time between observations of an identical source should exceed a certain interval.

**Fig. 1:**
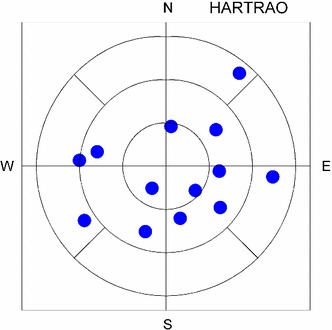
Example of a sky plot at station HARTRAO. The *plot* shows the observations in a time window of 1 h; 13 observed radio sources are distributed in nine segments

One possible method to define uniform sky coverage and to get a corresponding statistical number could be to divide the sky into segments and count those segments which contain an observed source in a certain time interval. In our case, the following rules are applied to count sky coverage, resulting in 13 sky segments (see Fig. 1):

o The sky above the antenna is divided in three different elevation segments: low (elevation >30$^\circ$), middle (elevation in the range of 30$^\circ$–60$^\circ$), and high elevation (higher than 60$^\circ$).

o The middle elevation segment is divided into four azimuthal segments and the low elevation segments into eight azimuthal segments.

If for one station the schedule has 13 observations per hour distributed in all 13 segments, the sky coverage is 100 %, i.e. this represents the best possible sky coverage. The worst case would be if all 13 observations are concentrated in one segment. The evaluation of the sky coverage can only be done for a certain time window, i.e. usually the time window of sky coverage should correspond to the sampling of the geodetic parameters. For instance, if zwd and clock parameters are estimated every hour, the time window of sky coverage should be 1 h, too. If the additional information about sky coverage is provided by the scheduling software, the analyst of the VLBI session will be able to choose the best time window for estimating zwd and clock parameters.

## New source-based scheduling strategy

The source-based approach implies that the scheduling program selects radio sources from the source catalogue independent of the sky distribution at individual stations (personal communications with Bill Petrachenko, Natural Resources Canada). With a better global station distribution and fast moving antennas, this simplification can be applied effectively for large networks with global coverage. This strategy automatically implies that different subnets are formed throughout the session to optimize geometry and number of observations. Thus, all possible baselines of the network are observed.

**Fig. 2:**
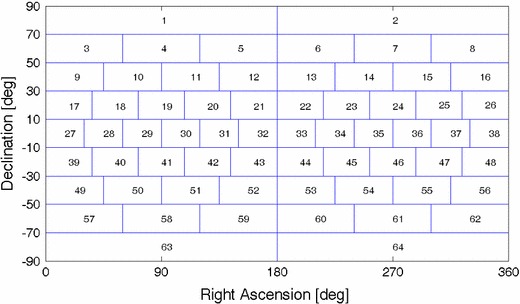
Grids on the celestial sphere

To evaluate the distribution of sources on the celestial sphere quantitatively, we followed a mathematical approach and split the celestial sphere into 64 segments by right ascension and declination (Fig. 2). To get segments with about equal area, the declination interval is set to 20$^\circ$, while the right ascension interval is a function of declination. For the statistics below, the segments which contain at least one observed source in a certain time interval are counted. If the schedule has 64 observed sources in a certain time span and these are distributed in all 64 segments, the distribution of sources on the celestial sphere is 100 %, i.e. the best possible distribution of sources. The worst case would be if all 64 sources are concentrated in one segment. In principle, this method is similar to that used to define the local sky coverage at stations. There are three options developed for the source-based scheduling strategy as described below.

### One source scheduled each time (abbreviated as 1-SAAT, i.e. 1 source at a time)

Only one radio source is considered for scheduling at each time epoch and as many stations as possible will participate in that scan. Hence, the subconfiguration consists of only one scan in this case. The segments are considered sequentially by their grid index, i.e. the loop from grid$_1$ to grid$_{64}$ is repeated in scheduling if the step size is set to one. If there is no suitable radio source in a particular cell, the next grid which contains suitable radio sources will be used as substitute. If the step size is two or four and the observed radio source of the previous scan is in cell$_i$, the next radio source to be observed is in cell$_{i+2}$ or cell$_{i+4}$. If there is more than one radio source in a selected cell, the second radio source is selected when this cell is re-visited. No more ranking and selection are used for the same cell when the source list is ordered using the three quality criteria of source structure, source strength and position stability. If there are several sources with similar quality in a cell, the errors related to one of them will tend to average out if we observe many of them. Furthermore, it is of advantage to observe a larger number of sources to improve the CRF.

### Two sources scheduled simultaneously (abbreviated as 2-SAAT, i.e. 2 sources at a time)

If a pair of radio sources is considered simultaneously, the two radio sources are as far apart as possible on the celestial sphere, i.e. they are located on opposite parts of the celestial sphere. Again, the step size can be one, two, or four. The whole network will be divided into two sub-nettings, observing the pair of radio sources simultaneously.

### Four sources scheduled simultaneously (abbreviated as 4-SAAT, i.e. 4 sources at a time)

When four radio sources are considered simultaneously, this is realized by the configuration of a regular tetrahedron. For example, if the first source has the declination of 90$^\circ$, the other three sources are in the same plane with a declination of $-$19.5$^\circ$ and they differ by 120$^\circ$ in right ascension. The step size can be one, two, or four. With a step size of four and source-based scheduling with four sources, the celestial sphere is fully covered with sources in a very short time. The whole network will be divided into four sub-nettings, each one observing one radio source simultaneously.

### Average visibility

In principle the n-SAAT strategy is possible, but we concentrate on the above three possibilities. In multi-source scheduling, if a station can observe more than one source at the given time with the 2-SAAT or 4-SAAT strategy, the solution is to observe the source which can improve the sky coverage at the station.

The average percentage of stations in a global network that can observe as a function of scheduling strategy was calculated at a fixed interval (i.e. 5 min) for a 24-h session, as seen in Table 1. Three different scheduling strategies and three different elevation angle cutoff values were tested. A global network consisting of 16 stations (see Fig. 9) was considered together with a fictitious catalogue of 64 radio sources that were homogeneously distributed on the celestial sphere. For the 1-SAAT configuration, less than half of the stations can observe one radio source simultaneously on average with a cutoff elevation angle of more than 5$^\circ$. And it can be found that the visibility varies largely related to the source position and the subnetting. Therefore, the option of a configuration with one source does not work properly for the global network. For the 2-SAAT configuration, the average percentage of stations that can observe simultaneously is decreased from 91.6 to 74 % with the cutoff elevation angle increasing from 5$^\circ$ to 15$^\circ$. For the 4-SAAT configuration, the average percentage of stations that can observe simultaneously is 100 % even when the cutoff elevation angle is as high as 10$^\circ$. And 99.99 % of the stations can observe at least one of the four sources if the cutoff elevation angle is set to 15$^\circ$. Please note that the 64 sources here are assumed to be distributed quite uniformly on the celestial sphere. Since the distribution of sources for geodetic VLBI with precise coordinates is non-uniform, i.e. most of them being in the northern hemisphere, the average visibility will be degraded slightly. When selecting 211 stable and compact sources from the ICRF2 as example, the average visibility of 4-SAAT is degraded from 100.0 to 99.96 % with the cutoff elevation angle of 5$^\circ$.

**Table 1:** Average percentage of stations in a global network that can observe as a function of scheduling strategy

Configuration	5$^{\circ }$ cutoff (%)	10$^{\circ }$ cutoff (%)	15$^{\circ }$ cutoff (%)
1-SAAT	45.8	41.4	37.0
2-SAAT	91.6	82.7	74.0
4-SAAT	100.0	100.0	99.990

A global network consisting of 16 stations was considered together with a fictitious catalogue of 64 radio sources that were homogeneously distributed on the celestial sphere. Three different scheduling strategies and three different elevation angle cutoff values were tested

## Fill-in mode (abbreviated as FI-mode)

By default, the scheduling program schedules one subconfiguration at each time epoch with either source-based or station-based strategy. A subconfiguration is a group of scans that could be scheduled at about the same time. A new scan will start as soon as possible, taking into account the time required for the antennas to move to the new source. A scan has to begin at the same time at all stations participating in the scan as required for correlation. When several stations observe together, several baselines can be formed. Each baseline observes for a different length of time to achieve the signal-to-noise ratio (SNR) target considering the sensitivities at the stations involved, i.e. some baselines require more observation time than others. Source flux densities and antenna sensitivities can be used, along with user-specified minimum SNRs, to compute scan times for each baseline. Furthermore, many limitations are imposed on the new scan and various conditions should be satisfied.

**Fig. 3:**
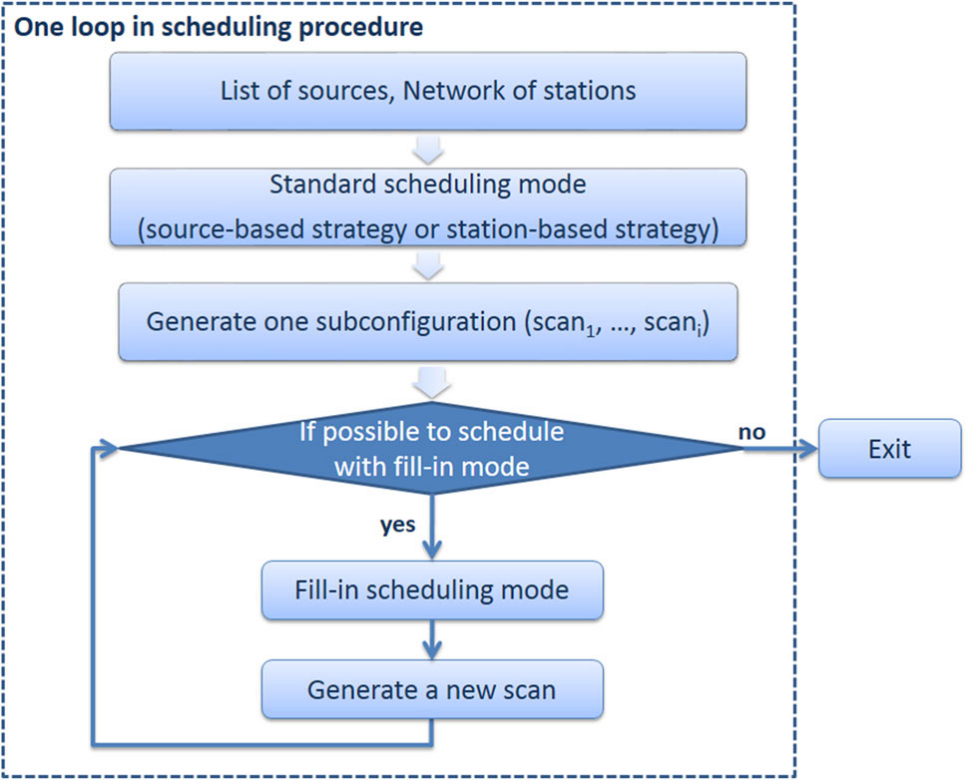
Flowchart illustrating the interaction between standard mode and FI-mode in the scheduling process

Thus, it is possible that there are at least two stations idling because of visibility, slewing limit, SNR limit, or other constraints. The FI-mode is introduced to reduce the amount of idling time, and it can be switched on in the local control file of Vie_Sched. When the scheduling program runs the FI-mode, only one radio source is considered at a time and only one scan is determined. Thus, the FI-mode is repeated until there are no more gaps. Figure 3 depicts the flowchart with standard mode and FI-mode in the scheduling procedure.

Considering the FI-mode, there are still a lot of possible scans ranked for observation. Vie_Sched attempts to schedule the scan with as many idling stations as possible as first and with the first end time as second criterion.

## Validation of the source-based scheduling strategy

To validate the newly developed source-based scheduling strategy, comparisons with the classical station-based scheduling strategy of Vie_Sched were carried out. Further comparisons were performed with the current scheduling software Sked (Gipson 2012), in which the sky coverage is one of the most important schedule optimizations. Sked was originally designed by Nancy Vandenberg and developed in the late 1970s at GSFC, and has been employed for scheduling VLBI observations for more than 30 years.

**Fig. 4:**
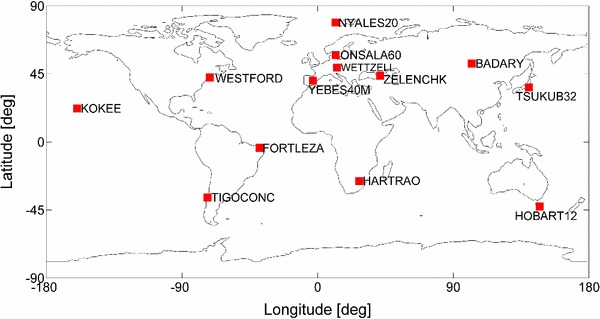
The 13-station CONT11 network

CONT11 was a campaign of continuous VLBI sessions employing 13 stations (see Fig. 4) and lasting 15 days during the second half of September 2011. The CONT11 schedules were prepared using the scheduling software Sked. For comparison purposes, we used the schedule file (c1101.skd) for the first CONT11 session. The second schedule was generated with Vie_Sched using the SB-FI strategy. The 2-SAAT-FI strategy in Vie_Sched was used to generate the third schedule. To be consistent with the original C1101 schedule file, the same station catalogue files, source parameters, and CONT11 frequency setup (512 Mbps data rate) were also employed in Vie_Sched. Hence, all the antenna parameters such as slewing rates, cable wrap, and system equivalent flux density (SEFD) represent the real antenna specifications. SNRs of 20 at X band and 15 at S band were used for all baselines, except those including the transportable, rather small (diameter 6 m) TIGO antenna which was scheduled with values of 15 and 12. The cutoff elevation angle of 5$^\circ$ was used. A minimum of 20 s and maximum of 200 s were set for scan length. The same source will not be observed within 30 min, and the time window of uniform sky coverage was set to 60 min. From the operational point, the time for the antennas to settle down and for calibration was 5 and 10 s, respectively. The time to allow the correlator to synchronize data was set to 3 s.

**Fig. 5:**
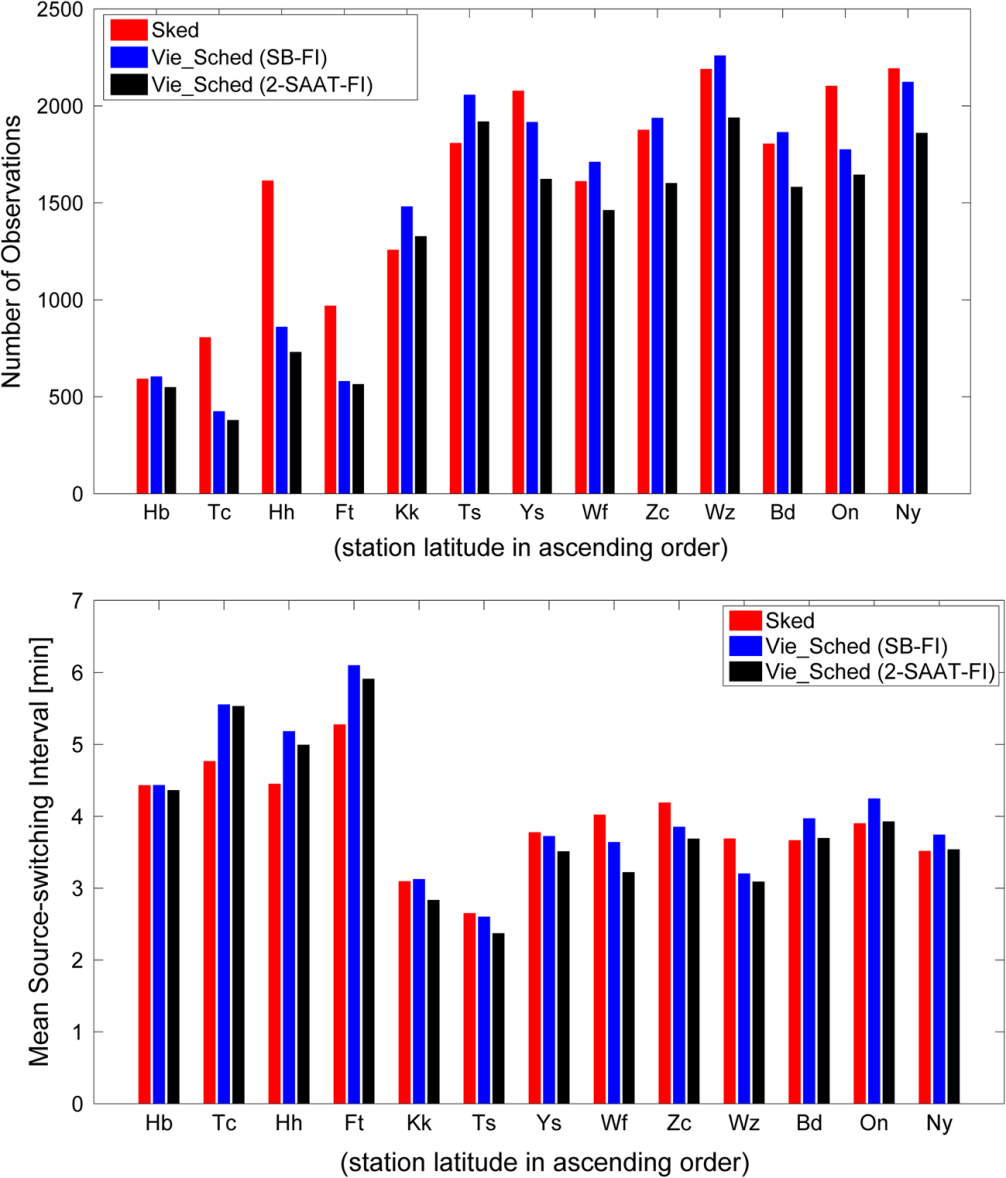
Distribution of observations (*upper plot*) and the mean source-switching interval (*lower plot*) at 13 stations of the CONT11 session

**Fig. 6:**
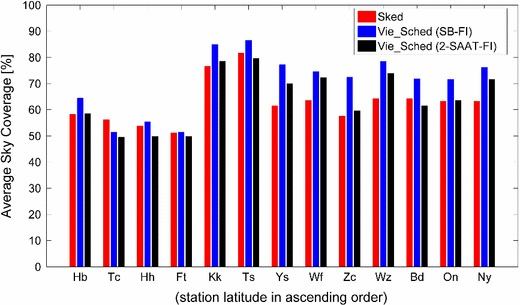
Average sky coverage during 1 h at 13 stations of the CONT11 session

Table 2 gives an overview of the key parameters of the above three schedules. The same pre-selection source list and source flux were used by Sked and Vie_Sched, while the number of observed sources was decided by the scheduling strategy. Figure 5 shows the number of observations and the mean source-switching interval during a 24-h session at individual stations. The source-switching interval refers to the time between the start of one scan and the start of the next scan (including on-source time, slewing time between sources, and even idling time). It is found that the number of observations begins to drop off along with the decrease of station latitude in all the schedules, as a consequence of the smaller subnet size in the southern hemisphere. Correspondingly, longer source-switching intervals at southern stations are obtained. It can be seen that Vie_Sched tends to have fewer observations for the southern stations. Sked has an option to preferentially select scans involving particular stations (personal communications with John Gipson). This is frequently used to make the distribution of scans across the stations more uniform. For CONT11 the preferred stations were TIGO (station Tc) and HARTRAO (station Hh). At the moment Vie_Sched is designed to give each station equivalent priority. Compared to the schedules from SB-FI scheduling strategy, more scans and less observations are obtained from the 2-SAAT-FI scheduling strategy, because the network is always divided into more subnets. The average sky coverage during 1 h obtained from the above three schedules is 62.7, 70.5, and 64.5 %, respectively, as shown in Fig. 6. Though the source-based scheduling does not optimize the local sky coverage at the individual sites, a good distribution of observed sources on the celestial sphere implies also a good sky coverage above the stations. Benefiting from the source-based scheduling strategy, more sources at individual stations are observed, especially at the northern stations.

**Table 2:** Statistics of the schedules for the CONT11 session

	Num of observed sources	Num of scans	Num of obs
Sked	103	1,196	11,344
Vie_Sched			
SB-FI	99	1,251	9,796
2-SAAT-FI	102	1,469	8,590

**Fig. 7:**
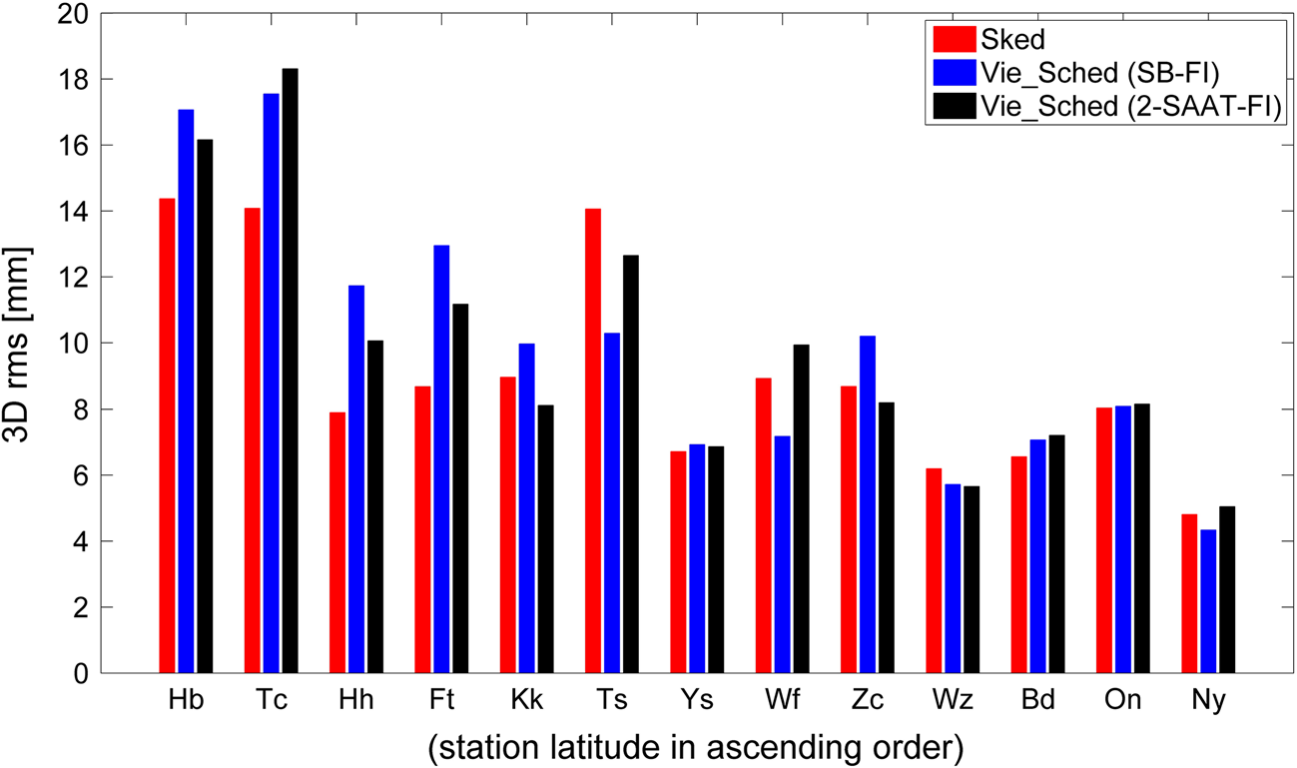
3D position rms values of station coordinates of the CONT11 session, estimated from the three different schedules

**Fig. 8:**
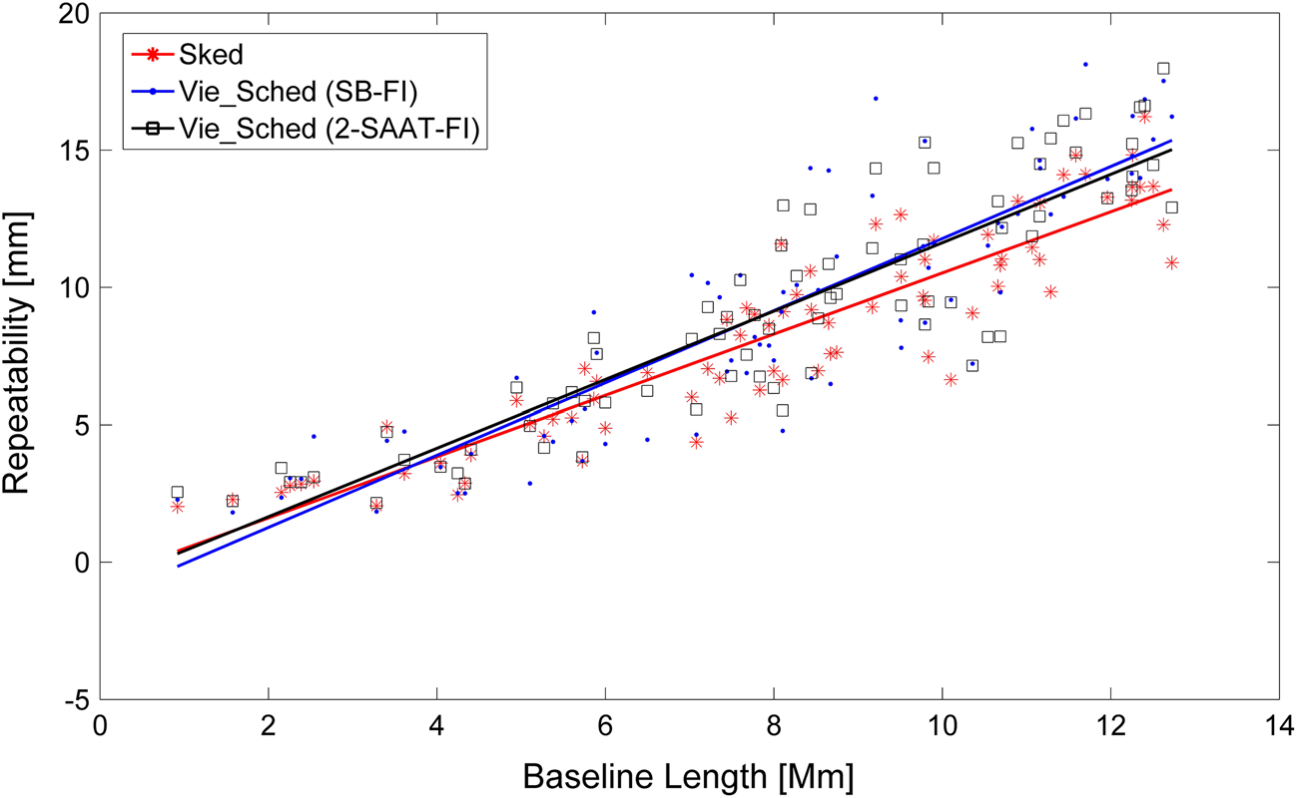
Baseline length repeatability of the CONT11 session, estimated from the three different schedules

Monte Carlo simulations (Pany et al. 2011) were employed to compare the geodetic parameters obtained from the above three schedules. Using the same 24-h schedule, 50 sessions were simulated with different realizations of stochastic errors. The same simulation parameters and estimation solution were used for the three schedules. The analysis was performed for each of the simulated files, and the sample of output parameters was analyzed statistically. For each station on each of the 50 days of simulated data, a three-dimensional (3D) position error was computed, which was the length between the apriori position and the estimated position. To obtain a single value for comparisons, the root mean square (rms) of 3D position error was computed over the 50 days for each station, as shown in Fig. 7. The primary quantity that was used throughout the simulation studies to characterize the performance is the mean of the rms 3D position errors for all the stations. The mean values of 3D rms position errors for the nine northern stations obtained from the three schedules are 8.1, 7.8, and 8.0 mm. And for the four southern stations, the mean values of the 3D rms position errors are 11.3, 14.8, and 13.9 mm. Figure 8 shows the comparisons of baseline length repeatabilities. The EOP results are presented in Table 3.

**Table 3:** Repeatability and formal uncertainty of EOP, the best value in each row highlighted

EOP parameter	Sked	Vie_Sched SB-FI	Vie_Sched 2-SAAT-FI
Repeatability ($\upmu$as, $\upmu$s)			
$x_\mathrm{p}$	**66.53**	92.14	70.31
$y_\mathrm{p}$	**49.68**	78.97	76.29
dUT	3.21	3.11	**3.04**
d*X*	29.10	33.54	**29.03**
d*Y*	26.64	**24.19**	29.14
Mean uncertainty ($\upmu$as, $\upmu$s)			
$x_\mathrm{p}$	**52.62**	66.17	60.67
$y_\mathrm{p}$	**44.42**	50.50	52.79
dUT	2.33	**2.11**	2.17
d*X*	17.31	**17.18**	19.22
d*Y*	17.92	**17.26**	18.87

$x_\mathrm{p}$ and $y_\mathrm{p}$ refer to polar motion in mas (microarcsecond), d*X* and d*Y* to celestial pole offsets in mas (microarcsecond), and dUT to UT1-UTC in ms (microsecond)

These comparisons confirm that the new scheduling software Vie_Sched with the source-based approach in particular provides schedules of high and comparable quality to those of Sked. In the next section, we will use it for the generation of VLBI2010 schedules.

## Analyses of the source-based scheduling strategy

To analyze the different scheduling strategies and modes, VLBI2010 schedules were generated. For the practical generation of the schedules, catalogues of suitable radio sources were required. As the basis for all the schedules in this section, a preliminary list of 211 suitable radio sources for geodetic VLBI was established here. The sources considered had positional accuracies of better than 200 $\upmu$as, their X-band structure index was lower than 3.0, and they were stronger than 0.25 Jy at both X and S bands (Sun 2013). It has to be noted that the characteristic values mentioned here represent a snapshot of the available data at the time this work was carried out (Ma 2009) and that these values may evolve with time. Furthermore, the characteristics of radio sources were still studied at dual bands (S/X band) because the complete data for the radio sources at wide VLBI2010 frequencies (2–14 GHz) are still unavailable now.

**Fig. 9:**
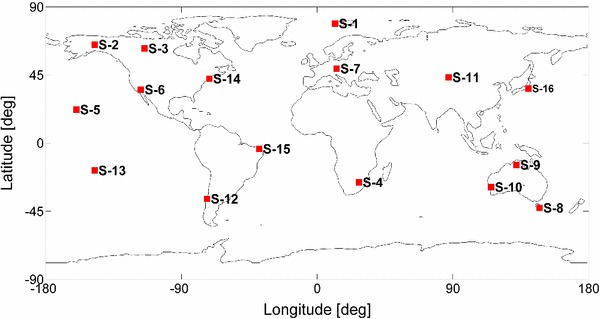
Test 16-station VLBI2010 network used for simulations

A VLBI2010 test network of 16 stations (see Fig. 9) was employed here and assumed to contain 16 identical antennas. The selection of stations was driven by a good coverage on all major tectonic plates and it should be noted that this network was only meant for test purposes. The antenna specifications are summarized below and correspond to the specifications of VLBI2010 antennas. The AZEL (azimuth-elevation) mount type was considered with an azimuth range of $-$270$^\circ$ to $+$270$^\circ$ and an elevation range of 5$^\circ$–88$^\circ$. The slew rate parameters were 12$^\circ$/s and 6$^\circ$/s in azimuth and elevation axes, respectively. The slew acceleration in both axes was 3$^\circ$/s^2^. The same SEFD of 2500 was used for all the antennas. The data rate was 8 Gbps (assuming bandwidth 128 MHz, sample rate 256 MHz, 16 channels, and 2 bits quantification).

The basic settings employed in the schedules were determined empirically and are summarized below. A minimum SNR of 20 and 15 (X/S band) was required to ensure successful detection of signals. The correlated source flux for each baseline was calculated and checked by the flux limit, which was also set to 0.25 Jy. A minimum of 5 s and maximum of 20 s were set for scan lengths. The cutoff elevation angle of 5$^\circ$ was used and the same source was not observed within 10 min. The time window to gain uniform sky coverage was set to 10 min. Four pairs of 24-h schedules were generated using different scheduling options. Within each pair the schedules differ only by whether FI-mode was turned on or off. The first two schedules were generated with the 1-SAAT strategy, the second pair of schedules with the 2-SAAT strategy, and the third pair with the 4-SAAT strategy. The last pair of schedule was generated with the SB strategy. The FI-mode was switched on and off for each pair of schedules, respectively. The various characteristics of the schedules are summarized in Table 4 and detailed explanations are given below.

**Table 4:** Characteristics of six options for the source-based scheduling and two options for the station-based scheduling

	1-SAAT	1-SAAT-FI	2-SAAT	2-SAAT-FI	4-SAAT	4-SAAT-FI	SB	SB-FI
Number of scans	3,531	5,790	5,611	7,225	9,088	10,329	4,824	6,410
Number of obs	86,939	149,865	129,470	131,207	82,411	85,121	125,148	137,185
Mean on-source time (s)	9.4	9.2	9.6	9.5	9.3	9.2	9.7	9.5
Mean slewing time (s)	10.7	11.1	11.4	11.4	12.8	12.7	13.5	13.3
Mean source-switching interval (s)	54.1	32.3	35.2	31.8	35.1	31.9	39.0	33.0
Mean sky coverage								
per 15 min (%)	71.9	83.3	83.2	85.2	89.1	91.0	93.8	96.5
per 30 min (%)	94.2	97.0	97.7	97.6	98.0	98.3	97.9	99.1
Distribution of sources								
per 15 min (%)	57.4	65.3	68.1	72.7	80.2	84.1	55.9	66.5
per 30 min (%)	88.1	88.7	89.3	89.6	89.2	89.6	67.8	77.0

When generating these schedules, it is found that the source-based approach is much faster than the station-based approach, because there are less computations required in source-based approach.

*Number of observations* Since the VLBI2010 network consists of fast moving antennas, the number of observations per hour is very large compared to that of today 10–20 scans/h. As expected, the 1-SAAT schedule gives the lowest number of scans. On the other hand, the 4-SAAT schedules give the largest number of scans, but less observations than the schedules that were prepared using the other strategies since the whole network is always divided into four subnets. The 1-SAAT-FI schedule shows great improvements with respect to the 1-SAAT schedule, since the FI-mode contributes with 39 % additional scans to the schedule. Comparing the 2-SAAT and 2-SAAT-FI schedules, slight improvements are achieved with the FI-mode (22 % additional scans are obtained). And for the 4-SAAT schedule, the FI-mode gives 12 % additional scans. For SB schedules, the FI-mode provides 25 % additional scans. Summarizing, the FI-mode plays an important role to decrease the idling time and to make use of stations efficiently, especially when the source configuration on the celestial sphere is poor.

*Mean source-switching interval* The on-source time, the minimum time required to ensure successful application of the delay technique, is based on the correlated flux of the observed source on each band at the baselines. With VLBI2010 antennas the on-source time is reduced to $<$10 s, assuming minimum SNR of 20 and 15 (X/S band). Furthermore, the fast-moving VLBI2010 antennas allow that each sky position can be reached in 20 s. Statistics reveal that longer inter-scan slewing times are required for the 4-SAAT and SB strategies. The average source-switching interval was also calculated for each schedule and summarized in Table 4.

**Fig. 10:**
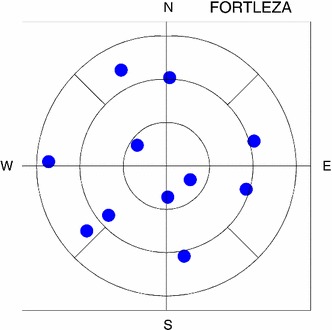
Sky plot at station FORTLEZA from the 4-SAAT-FI schedule. The *plot* shows the observations in a time window of 6 min: 11 observed radio sources are distributed in nine segments

*Sky coverage at stations* Table 4 reveals that better sky coverage is obtained with the 4-SAAT and SB strategies, even though the slewing times are longer on average. For example, Fig. 10 shows the sky plot at station FORTLEZA in a time window of 6 min.

*Distribution of observed sources on the celestial sphere* The best distribution of observed sources on the celestial sphere is obtained from the 4-SAAT and 4-SAAT-FI strategies. Therefore, the 4-SAAT has great advantages in providing a good source distribution on the celestial sphere.

## VLBI2010 simulations

### Simulation parameters

In this section, Monte Carlo simulations were employed to study the relationship between the schedules and the final products of geodetic parameters. The studies focus on the investigations of the impact of source-based scheduling optimization criteria on the accuracy of estimated station positions, baseline length repeatabilities, and the EOP.  Here, only four scheduling options with FI-mode were employed for the simulations, i.e. in the following order 1-SAAT-FI, 2-SAAT-FI, 4-SAAT-FI, and SB-FI.

The new scheduling package (Vie_Sched) is directly connected to the other packages of VieVS and the simulation studies are realized by running a sequence of different packages of VieVS. Simulated VLBI observables were generated taking into account the three most important stochastic error sources in VLBI, i.e. wet troposphere delay, station clock, and measurement error Wresnik et al. 2008; Pany et al. 2011. To simulate the wet delays as realistically as possible, the turbulence theory Treuhaft 1987 with a dedicated strategy proposed by Nilsson and Haas (2010) was applied. The wet delays following turbulence theory took into account the covariance information between all observations at a station and a particular series of equivalent zwds was triggered by random numbers. The turbulent troposphere was modelled using site-dependent structure constants $C_\mathrm{n}$Nilsson et al. 2009, effective wet heights $H$, and components of wind velocity ($V_\mathrm{n}$ and $V_\mathrm{e}$). The zwd at the beginning of the time series ($\mathrm{zwd}\_0$), the correlation interval ($\mathrm{d}h_\mathrm{seg}$), and the height increment for the numerical integration ($\mathrm{d}h$) were set to standard values. Stochastic errors of station clocks can be simulated as the sum of random walk and integrated random walk stochastic processes (Herring et al. 1990). Our simulations were performed with power spectral densities corresponding to Allan Standard Deviations (ASD) of $1\times 10^{-14}$ at 50 min, which is a typical frequency stability of current H-masers. The contribution of the measurement error to the simulated delay observables is small compared to the that of troposphere and clock. A typical measurement precision for today’s VLBI system is 10–30 ps (Schuh and Behrend 2012), while VLBI2010 is aiming at a measurement error as low as 4 ps by significantly increasing the data rate and the recording bandwidth (Petrachenko et al. 2009a). In our VLBI2010 simulations, a white Gaussian noise (WN) with a standard deviation of 8 ps was used to represent all system errors. The simulation parameters are summarized in Tables 5 and 6.

**Table 5:** Simulation parameters

$H$ (m)	2,000
$V_\mathrm{n}$ (m/s)	0.00
$V_\mathrm{e}$ (m/s)	8.00
$\mathrm{zwd}\_0$ (mm)	250
$\mathrm{d}h\mathrm{seg}$ (h)	2
$\mathrm{d}h$ (m)	200
clock ASD	10$^{-14}$@50 min
WN (ps)	8

**Table 6:** Site-dependent $C_\mathrm{n}$ in $\mathrm{m}^{-1/3}$

Station	$C_\mathrm{n}$ $\times$ 10$^{-7}$	Station	$C_\mathrm{n}$ $\times$ 10$^{-7}$
S-1	0.65	S-9	1.68
S-2	1.16	S-10	1.76
S-3	1.24	S-11	1.79
S-4	1.34	S-12	2.08
S-5	1.39	S-13	2.19
S-6	1.45	S-14	2.30
S-7	1.50	S-15	2.46
S-8	1.60	S-16	3.45

For the Monte Carlo simulations, 50 sessions were simulated using the same 24-h schedule but different realizations of noise delays, each time creating new values for zwds, clocks, and white noise. The analysis was performed for each of the simulated files, and the sample of output parameters was analyzed statistically.

### Estimated parameters

In the least-squares parameter estimation part of VieVS, most of the estimated parameters are modelled by piecewise linear offset functions (Teke et al 2009). The main goal of the estimation process in the simulations was to investigate the impact of scheduling strategies on the estimates of geodetic parameters. The parameters to be estimated were troposphere parameters, clock parameters, and station positions, as well as daily EOP.

1. The tropospheric slant wet delays were estimated as piecewise linear zwds and superimposed gradients as proposed in the IERS Conventions 2010 (Petit and Luzum 2010). The time interval between zwd piecewise linear offsets was 15 min, and a relative constraint of 15 mm implied that adjacent offsets were identical to a standard deviation of 15 mm. The same held for the gradients, where gradients after 30 min were assumed to be identical with a standard deviation of 0.5 mm. The absolute constraints of 1 mm were applied on the gradient offsets, i.e., additional observation equations were added which implied that the gradient offset was zero ${\pm }1$ mm standard deviation.
2. Station clocks were modelled with a second-order polynomial superimposed with piecewise linear offsets every 60 min which were constrained with 13 mm relatively.
3. The components of station position were treated as offsets and were estimated once per 24-h session. No-net-rotation (NNR) and No-net-translation (NNT) conditions were employed for all apriori station coordinates.
4. The EOP offsets were also estimated once per 24-h session.
5. No source coordinates were estimated.

### Results

The following criteria were used to evaluate the potential of the VLBI2010 system in the simulations: the rms of the 3D station position residuals, formal errors and standard deviations of the EOP.

**Fig. 11:**
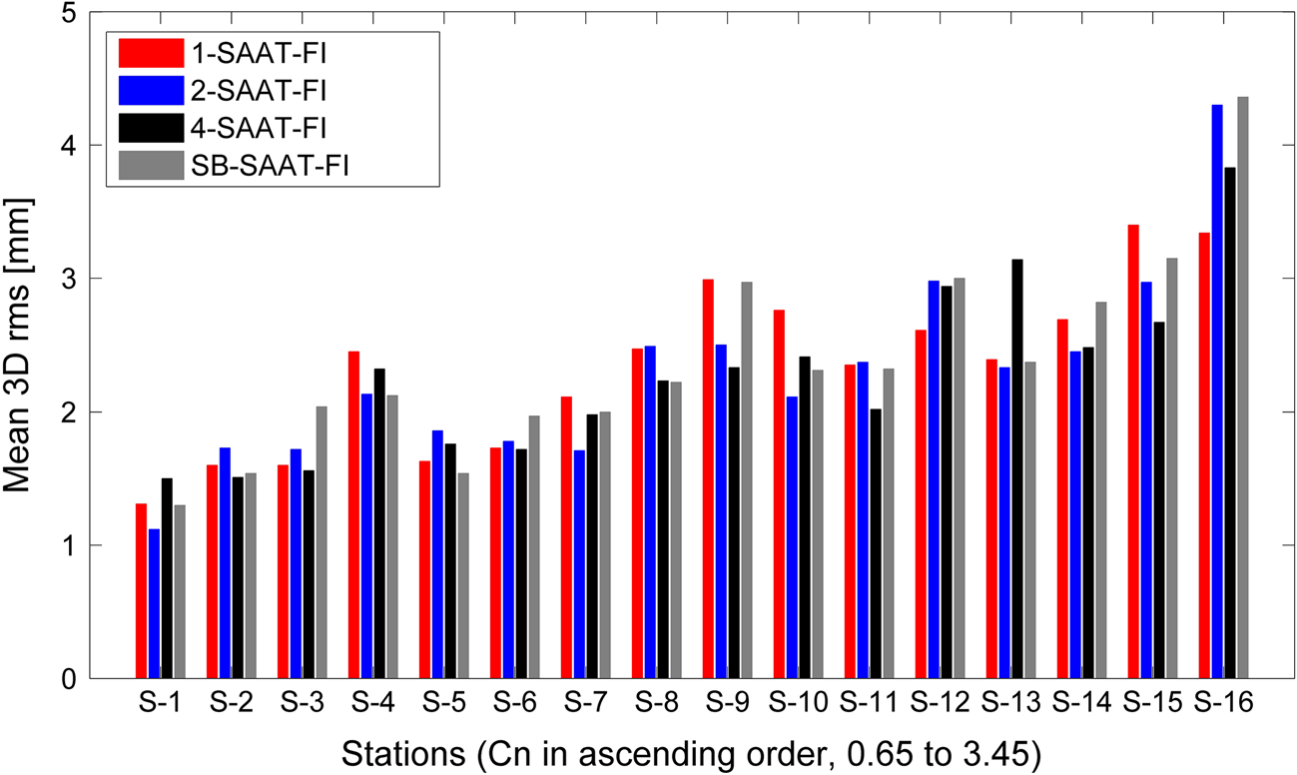
3D position rms values of station coordinates of the VLBI2010 session, estimated from the four different schedules

*Station position estimation* Figure 11 shows the rms of 3D position errors for all the 16 stations. The mean values obtained from the four schedules are 2.34, 2.28, 2.27, and 2.38 mm. The baseline length repeatabilities are also found to be very similar for all these schedules. From Fig. 11, it is also evident that the large values for the 3D station rms are related to the refractive index structure constant $C_\mathrm{n}$ since the wet troposphere is a very important error source for the geodetic products. More accurate results of geodetic VLBI will be obtained when the model of the troposphere wet delay is significantly improved and/or the number of observations at the site is dramatically increased.

*EOP estimation* Table 7 shows the repeatability and formal uncertainty of the Earth orientation parameters (polar motion, UT1-UTC, and nutation) estimated per 24-h session from the four different schedules. The EOP derived with the different scheduling options are of similar quality. No clear superiority of one option can be seen and the scheduling strategies reach the same level of accuracy.

**Table 7:** Repeatability and formal uncertainty of EOP, the best value in each row highlighted

EOP parameter	1-SAAT-FI	2-SAAT-FI	4-SAAT-FI	SB-FI
Repeatability ($\upmu$as, $\upmu$s)				
$x_\mathrm{p}$	6.83	7.33	6.98	**6.19**
$y_\mathrm{p}$	**6.98**	8.11	7.18	7.90
dUT	0.41	**0.34**	0.35	0.40
d*X*	7.30	7.92	**5.33**	6.22
d*Y*	7.04	5.91	**5.49**	5.75
Mean uncertainty ($\upmu$as, $\upmu$s)				
$x_\mathrm{p}$	2.49	**2.45**	2.90	2.62
$x_\mathrm{p}$	2.34	**2.32**	2.74	2.46
dUT	**0.14**	**0.14**	0.17	0.16
d*X*	**2.04**	2.08	2.47	2.20
d*Y*	**2.03**	2.05	2.40	2.17

$x_\mathrm{p}$ and $y_\mathrm{p}$ refer to polar motion in $\upmu$as (microarcsecond), d*X* and d*Y* to celestial pole offsets in $\upmu$as (microarcsecond), and dUT to UT1-UTC in $\upmu$s (microsecond) $C_\mathrm{n}$ in $\mathrm{m}^{-1/3}$

From the above simulations, the geodetic products from the four schedules are quite close to each other. However, we can also find that the strategy with 2-SAAT or 4-SAAT plus FI-mode is slightly better for future VLBI2010 networks, in terms of repeatabilities of station coordinates, compared to the station-based strategy.

## Summary and outlook

We have shown in simulations that the new scheduling package Vie_Sched as a part of the VieVS provides schedules for the determination of geodetic parameters with an accuracy comparable to existing scheduling software. Furthermore, we have demonstrated that Vie_Sched will be well suited for VLBI2010 observations with significantly more stations and observations. The new scheduling package offers the possibility to optimize the schedules for various parameters and different criteria. The source-based scheduling strategy uniformly fills the celestial sphere with observations and—as a side effect—also creates uniformly distributed observations at the stations. The 4-SAAT strategy is suggested for the future VLBI2010 network (up to 30 stations), and the 2-SAAT strategy and station-based strategy for the current IVS network (around 8–10 stations). The 1-SAAT strategy and station-based strategy are suitable for the regional network. In Vie_Sched, the number of calculations necessary for the source-based scheduling strategy is clearly reduced compared to the conventional station-based approach, which is especially advantageous for global and large VLBI2010 networks.

One of the major challenges for Vie_Sched in future is the scheduling of mixed observations, i.e., broadband observations on the one hand and classical S/X observations on the other hand. While the largest amount of observations can be scheduled with separate networks, some linking observations will be necessary to connect the frames.
